# Assessment of mycotoxin sequestration efficacy in *Saccharomyces cerevisiae* by-products cultured in wheat bran and whey protein medium

**DOI:** 10.1038/s41598-024-53633-9

**Published:** 2024-02-07

**Authors:** Pardis Sadat Mirseyed, Shahpour Kheirabadi, Rojin Anbarteh, Morteza H. Ghaffari

**Affiliations:** 1 Protein Research Center, Shahid Beheshti University, Tehran, Iran; 2 Department of Animal Science, College of Agriculture and Natural Resources, University of Tehran, Karaj, Alborz Iran; 3 Antimicrobial Resistance Research Center, Institute of Immunology and Infectious Disease, Iran University of Medical Sciences, Tehran, Iran; 4 Institute of Animal Science, University of Bonn, 53115 Bonn, Germany

**Keywords:** Biochemistry, Health care

## Abstract

Mycotoxins are metabolic products of fungi found in feed for farm animals and pose a major threat to food safety due to their adverse health effects. The development of strategies to reduce their bioavailability is crucial. In this context, the cell wall components of *Saccharomyces cerevisiae* (YCW), especially β-d-glucans and Mannan-oligosaccharide, have been recognized as potent mycotoxin binders. The objective of this research was to develop a novel culture medium to increase the biomass yield of *S. cerevisiae* and optimize cell disruption by stepwise physical lysis and hydrolytic preconditioning. This process resulted in a yield of approximately 56% reducing saccharides and 28.54% protein. Subsequently, the β-glucan was extracted after cell wall sequestration. The isolated YCW and extracted β-glucan were characterized both individually and synergistically to evaluate their antibacterial properties and analyze their Fourier transform infrared (FTIR) spectra. In vitro evaluation of antibacterial activity revealed that a concentration greater than 250 μg/mL of YCW-β-glucan blend significantly inhibited the growth of Gram-negative bacteria. In addition, this blend showed good adsorption of various mycotoxins, including *Aflatoxin B1*, *Ochratoxin A,* and *Zearalenone,* the latter of which exhibited a remarkable adsorption rate of 80.85%. This study highlights the promising potential of a combination of YCW and β-glucan as a robust strategy to address the pervasive problem of mycotoxin contamination in feed.

## Introduction

The spores of fungal species including mold are minute, therefore they naturally spread around almost everywhere, which makes their eradication burdensome. Mycotoxins, secondary metabolites of fast-growing fungi, are defined as microscopic toxic stable molecules under varying environmental conditions. Environmental challenges, such as meteorological events, unsuitable conditions for feed products, and poor plant health, can lead to increased growth and promotion of fungal species^1^. Cereals and plant foodstuffs consumed by both animals and humans are susceptible to contamination by over 400 known mycotoxins. Among these are aflatoxins, *Ochratoxin A*, and *Fusarium* toxins, all of which have been identified as major contaminant^2^. Aflatoxins are toxic byproducts of fungi metabolism, primarily *Aspergillus* species like *A. flavus* and *A. parasiticus*, contaminating agricultural products. Bianco et al. reported that *Aflatoxin B1* (AFB1), the ubiquitous Aflatoxins type, can manifest anti-proliferative activity on macrophages, disrupting the proper function of innate immune system in animals and humans^3^. Recognizing its carcinogenic properties, the International Agency for Research on Cancer (IARC) has classified it as a human carcinogen. Similarly, *Ochratoxin A* (OTA), from *Penicillium* and *Aspergillus*, is a potential human carcinogen. This chlorinated isocoumarin derivative can amide-bond with feed components^4–7^. *Zearalenone* (ZEN), a *Fusarium* byproduct found in cereals. This compound has a relatively weak acidity characterized by the presence of a di-phenolic group, resulting in a pKa of about 5. It has more hydrophobic than AFs and bears a surface negative charge^2,3,8^. Given the significant economic and health implications of mycotoxin contamination, it is imperative to adopt preharvest and postharvest strategies to protect vulnerable animal feed. Preventive measures involve resistant crops, fungicides, preservatives, and Good Agricultural Practice compliance. Post-harvest interventions encompass temperature control, irradiation, cold plasma, and chemical detoxification techniques, including ozonation^9,10^. However, a cost-effective alternative to mitigate mycotoxicosis and reduce mycotoxin bioavailability entails employing absorbents in animal feed^11^. This approach binds mycotoxins through various interactions like hydrophobic, hydrogen, electrostatic, and coordinative bonds, thereby reducing their absorption in the digestive tract. Incorporating specific absorbents like aluminosilicates, activated carbon, silica, and clays in animal diets has proven effective in minimizing mycotoxin absorption and mitigating their detrimental effects on animal health^2,12^. By attaching to mycotoxins as they move through the digestive system, adsorbents lessen the likelihood of these harmful toxins reaching and damaging the tissues or overall health of the animal^13^. Microorganisms, specifically *S. cerevisiae* and its polysaccharides, have emerged as potent toxin binders, garnering significant global research interest^6,12^. The yeast cell wall (YCW) predominantly consists of polysaccharides, such as β-D-glucans, Mannanoligosaccharides, and chitin, which constitute 85–90% of its structure. These YCW polysaccharides have demonstrated a capacity to absorb 32–90% of mycotoxins, contingent upon the toxin concentration^7,12^. The β-d-glucan content significantly influences YCW's absorption capacity, while chitin restricts toxin access to β-d-glucans. By optimizing the culture medium, the chitin content can be reduced, thus enhancing toxin absorption in YCW^14^. Extracted β-d-glucans have proven particularly effective in absorbing non-steroidal mycotoxins like ZEN due to their three-dimensional structure and insolubility. These polysaccharides counteract microbial infections without competing with immune system stimulation, and unlike mineral absorbents, they are biodegradable^7,14^. The objective of this study is to develop an optimized culture medium to increase the yield of YCW biomass. Our approach focuses on enhancing polysaccharide synthesis through advanced disruption techniques and refining the β-glucan extraction process. The study further aims to characterize the extracted β-glucan and YCW using Fourier transform infrared (FTIR) spectroscopy and antibacterial assays, and to assess their effectiveness in mycotoxin binding and elimination.

## Material and methods

### Chemical agents and yeast strain

*Saccharomyces cerevisiae* variant (SSL-3) was obtained from Iranian Biological Resource Center (IBRC) and stored at − 80 °C until needed for the experiments. Flavourzyme^®^ 2.4 L FG (CAS: 9001-92-7), manufactured by Novozymes, Denmark, was also purchased. All experiments were performed with reagent grade materials. The study complies with ARRIVE guidelines for reporting in vivo experiments and all methods were performed following the relevant guidelines and regulations. Experimental research and field studies on plants (either cultivated or wild), including the collection of plant material, must comply with relevant institutional, national, and international guidelines and legislation.

### Growth of yeast cells

A series of experiments, including the cultivation of *S. cerevisiae* cells in three types of culture media were conducted. Before inoculating the yeast cells in the main medium, pre-cultivation was performed. In this phase, the SSL-3 strain was propagated in Erlenmeyer flasks of 250 mL capacity containing 50 mL of YPG medium composed of 2% glycerol, 2% bactopeptone, and 1% yeast extract (w/v). The pH of the medium was adjusted to 6.5 before incubation at 37 °C and a shaking speed of 120 rpm for 24 h. The primary culture medium, composed of lignocellulosic ingredients "Wheat bran", was prepared using a two-step acid hydrolysis process. The first phase involved the breakdown of lignin bonds to extract cellulose and hemicellulose. The second phase facilitated the recovery of glucose from these components. This is crucial as yeast cells can utilize glucose as a carbon source^13^. In the first phase, a solution of 10% wheat bran (w/v) was prepared in 1000 mL of sterilized water, then 2% sulfuric acid 1N (w/w) was added. This mixture was autoclaved at 130 °C for 20 min, subsequently filtered using Whatman paper, and the pH was adjusted to 6.5 with 1N NaOH. The extract was then centrifuged to eliminate impurities at 5000 rpm for 10 min. In the second phase, the supernatant was treated with 1% (w/w) of 1N sulfuric acid, and acid hydrolysis was carried out in a manner like the previous step. Finally, the preliminary culture medium was used to inoculate the primary cultures, applying a volume fraction of 5% to achieve an optical density (OD) of 0.2 at 600 nm. This ensured the commencement of each experiment with an appropriately dense cell population in the correct growth phase. Post-preparation, the main cultures were incubated at 37° for 72 h. The preparation parameters for each culture medium are detailed in Table 1.

**Table 1:** Parameters of preparation of cultures medium experimental system.

Cultivation medium	Preparation in 1000 mL deionized water	Abbreviation^a^
YPG	Yeast extract 1% (w/v), bactopeptone 2% (w/v), glycerol 2% (w/v)	YPG
Wheat bran/Yeast extract	Wheat bran 10% (w/v), yeast extract 1% (w/v), MgSO_4_. 7H_2_O 0.25% (w/v)	(Wb/Ye)
Wheat bran/Whey protein	Wheat bran 10% (w/v), whey protein 1% (w/v), MgSO_4_. 7H_2_O 0.25% (w/v)	(Wb/Wp)

^a^Abbreviation of a given method.

To assess and contrast the biomass yield across the different growth mediums, the OD at 600 nm was measured at 24, 48, and 72-h intervals post-incubation, using a µQuant spectrophotometer from BioTek, USA.

### Methodology of yeast cell wall preparation using selected techniques

Various methods were used to disrupt the optimally cultured yeast cells in the three different experimental systems (Table 2).

**Table 2:** Parameters of the experimental system for the preparation of yeast cells.

Experimental system	Cell suspension in deionized water	Abbreviation^a^
Hydrolysis	pH 6.5, 55 °c, 24 h, 180 rpm	H
Physical lysis	pH 6.5, 55 °c, 24 h, 220 rpm	Ph
Hydrolysis-Physical lysis	pH 6.5, 55 °c, 12 h, 180 rpm 55 °c, 12 h, 220 rpm	H-Ph

^a^Abbreviation of a given method.

### Hydrolysis (H)

In this method, yeast cells were disrupted by enzymatic hydrolysis. A 16% (w/v) suspension of *S. cerevisiae* cells was prepared using distilled water. The pH was adjusted to 6.5 before adding 0.5% (w/v) Flavourzyme (Novozymes). The mixture was then incubated at 55 °C for 24 h while stirring. To stop enzymatic activity, the mixture was heated to 90 °C for 10 min after hydrolysis.

### Physical lysis (Ph)

A 16% (w/v) suspension of *S. cerevisiae* cells in distilled water was prepared, with the pH adjusted to 6.9. This was then lysed using 1.0 mm diameter glass beads, which constituted 40% of the working volume of the vessel. This physical lysis was carried out over a period of 24 h at 55 °C with agitation.

### Enzymatic pretreatment followed by physical lysis (H-Ph)

First, enzymatic pretreatment was performed by adding 0.25% (w/v) Flavourzyme to a 16% (w/v) *S. cerevisiae* cell suspension and shaking it at 55 °C for 12 h. Enzyme activity was then stopped by heating the vessel to 90 °C for 10 min. After the pH was adjusted to 6.9, glass beads were added to the hydrolyzed suspension, accounting for 40% of the working volume of the vessel, followed by 12 h physical lysis process at 55 °C with agitation.

### Evaluation of the disruption of the yeast cells

#### Microscopic images

To evaluate the efficacy of each disruption method, disrupted yeast cells were stained with a 0.1% methylene blue solution before and after disruption and examined under a light microscope. Observations were made at 100× magnification.

#### Analysis of yeast cell disruption

The effects of cell disruption were studied in two different experiments. (1) Viability assay: 48 h after cell disruption, the viability of disrupted yeast cells was determined by growing yeast suspensions on YPG agar plates (consisting of 0.5 g/L yeast extract, 1.2 g/L bactopeptone, 1.5 g/L agar, and 2% glycerol). Images were acquired after 24 h incubation period at 37 °C. (2) Turbidity measurement: the turbidity of the supernatant before and after disruption was measured at OD 260 and OD 280 nm, indicating the release of nucleic acids and proteins, respectively. This was done after centrifugation of the suspension (8000 rpm for 10 min) and dilution of the supernatant with water. Water served as a blank in this measurement.

#### Estimation of the total nitrogen content

After disruption, the insoluble cell wall was precipitated by centrifugation (5000 rpm for 10 min) and rinsed twice with distilled water. The nitrogen content in the accumulated cell walls was determined by the Kjeldahl method, and the results were calculated using a conversion factor of 6.25 per crude protein^15^.

#### Total saccharide content

The total saccharide content was determined by the colorimetric method (DNS) at a wavelength of 543 nm (Analytikjana-Specord 200/Germany). The DNS reagent was prepared according to the method of Lindsay. For this purpose, a solution of 1% (w/v) 3,5-dinitrosalicylic acid, 20% (v/v) NaOH 2 M and 50% (v/v) sodium potassium tartrate was prepared in a 500 mL volumetric flask and made up with distilled water. The reagent solution was then stirred overnight. The cell wall samples were mixed 1:4 with the DNS reagent and the solutions were placed in a hot water bath for 5 min and then quickly cooled on ice. The absorbance was then measured. Sucrose content was calculated using a standard curve constructed for glucose (y = 0.6654x + 0.0216, R^2^ = 0.997).

### Production of β-glucan with optimized cell wall

The analyses conducted to determine the most effective method of yeast cell disruption for obtaining a cell wall rich in polysaccharides and low in protein led to the performance of β-glucan extraction using the following method. To extract β-glucan from the optimized YCW (H-Ph), 200 mL of a 0.1 N sodium hydroxide (NaOH) solution was prepared. To initiate the extract, 16 g of YCW was boiled in the NaOH solution in an Erlenmeyer flask for 15 min with stirring. After cooling for about 1 h, the mixture was centrifuged (5000 rpm for 5 min). The supernatant was discarded after centrifugation and the residue was stored. After preparation of 0.1 N hydrochloric acid (HCl), acid extraction was performed with the resuspended residue in 200 mL of 0.1 N HCl solution and boiled for 15 min while stirring. After cooling, the pellet was harvested by centrifugation (5,000 rpm for 5 min). To neutralize the residue, it was boiled with 200 mL ethanol solution at 80 °C. After boiling the ethanol, the pellet was centrifuged at a higher speed (7000 rpm for 5 min) and washed twice with distilled water. Finally, the extracted β-glucan was dried for 24 h at 50 °C.

### Fourier transform infrared spectroscopy (FTIR)

To evaluate the structure of the optimized YCW and extracted β-glucan from that, the FTIR method was performed. The infrared spectra of native preparations were measured in the wavelength range of 4000–400 cm^−1^ using an ATR FTIR (Nexus 470, USA) at room temperature.

### Antibacterial activity

The antibacterial properties of the optimized YCW, β-glucan, and their combination (with a ratio of 2:1 for YCW and β-glucan, respectively) were investigated using the agar well diffusion and macrodilution methods on Gram-negative *E. coli* (ATCC 25922) and Gram-positive *S. aureus* (ATCC 25923). In the agar well diffusion assay, the desired bacteria were cultured on Muller Hinton Agar (MHA) and incubated for 24 h, reaching a concentration of 0.5 McFarland (1.5 × 10^8^ CFU/mL). Each microorganism (10^6^ CFU/mL) was inoculated onto the surface of the MHA plates. The wells were placed on the agar plates using a sterile Pasteur pipette (diameter 6 mm). 100 µL of the desired material was poured into the wells. Finally, all plates were incubated at 37 °C for 18–24 h and the zones of inhibition were measured.

To determine the minimum inhibitory concentration (MIC) and minimum bactericidal concentration (MBC), the macrodilution method was used. For this purpose, 6 tubes containing 1 mL of autoclaved MHB culture medium were prepared. Then, 1 mL of the desired substance was added to the first tube. Then the dilution series was prepared. In the next step, 1 mL of bacterial suspension (10^5^ CFU/mL) was added to each of the tubes. The solution with the inoculated bacteria in MHB without the desired substance and the solution with the desired substance without bacteria were considered as negative and positive controls, respectively. Finally, all tubes were placed in a shaking incubator at 37 °C and 120 rpm for 24 h. After MIC determination, 100 µL of the cultures that showed no visible growth were cultured on the MHA plates and incubated at 37 °C for 24 h. The lowest concentration of a substance that killed 99.9% of the bacteria was considered the MBC endpoint.

### In vitro capacity of binders to form complexes with various toxins

An in vitro procedure was performed to estimate the complex ability of AFB1, OTA, and ZEN toxins with binders. Optimal YCW individually and a mixture of YCW and β-glucan were used as absorbents. The ability of absorbents and toxins to form complexes was studied according to the method of Hojati et al.^11^. To prepare a standard solution of the above toxins, 5 mg of each toxin was prepared in 3 mL of acetonitrile and added to 500 mL of distilled water. Each sequestering agent (1 g) was placed in a 125 mL Erlenmeyer flask containing 100 mL of 10% methanol and mixed with a magnetic stirrer for 1 h to obtain the stock solution. In the next step, 5 mL of the stock solution was added to the 0.5% suspension of each separating agent and each sample was incubated at 39 °C for 2 h with shaking at 200 rpm. After incubation, the mixtures were centrifuged (7000*g*, 15 min) and the supernatant was used to analyze the residual unbound toxins by high-performance liquid chromatography (HPLC) (Perkin Elmer, Boston, USA). The unbound residual toxins in the supernatants were analyzed using an Ace 5 μm C18 column (250 mm × 4.6 mm), an Ace 5 C18 guard column (Phenomenex Grace Vydac, USA), a loop of 50 μL, at a flow rate of 1 mL/minute, at room temperature, with water: acetonitrile: glacial acetic acid (99:99:2, v/v/v) as the mobile phase and fluorescence detection (λ-excitation^OTA^ = 330 nm, λ-emission^OTA^ = 460 nm), (λ-excitation^AFB1^ = 360 nm, λ-emission^AFB1^ = 440 nm), (λ-excitation^ZEN^ = 274 nm, λ-emission^ZEN^ = 440 nm).

### Statistical analysis

Data were analyzed using the GLM procedure (SAS 9.4, 2014) to model the fixed effects of treatment, according to the following model:

$${\text{Y}}_{{{\text{ijk}}}} = \mu + {\text{S}}_{{\text{i}}} + {\text{L}}_{{\text{j}}} + \left( {{\text{SL}}} \right)_{{{\text{ij}}}} + \varepsilon_{{{\text{ijk}}}} .$$

where Y_ij_ is the dependent variable, μ is the overall mean, S_i_ is the fixed effect of source of supplemented fat, L_j_ is the fixed effect of levels of supplemented fat, (SL)_ij_ is the interaction effect of source and levels of fat, and ε_ij_ is residual error associated with each Y_ij_. Least squares means with a significant F-test (P < 0.05) were compared using PDIFF of SAS. Significance was assessed using Least Squares Means with an F-test (P < 0.05), and trends noted for 0.05 < P < 0.10. The study included six repetitions per condition within the experiment to enhance data reliability. Error bars in graphs and tables represent standard errors.

## Result and discussion

### Yeast cell cultivation

**Figure 1:**
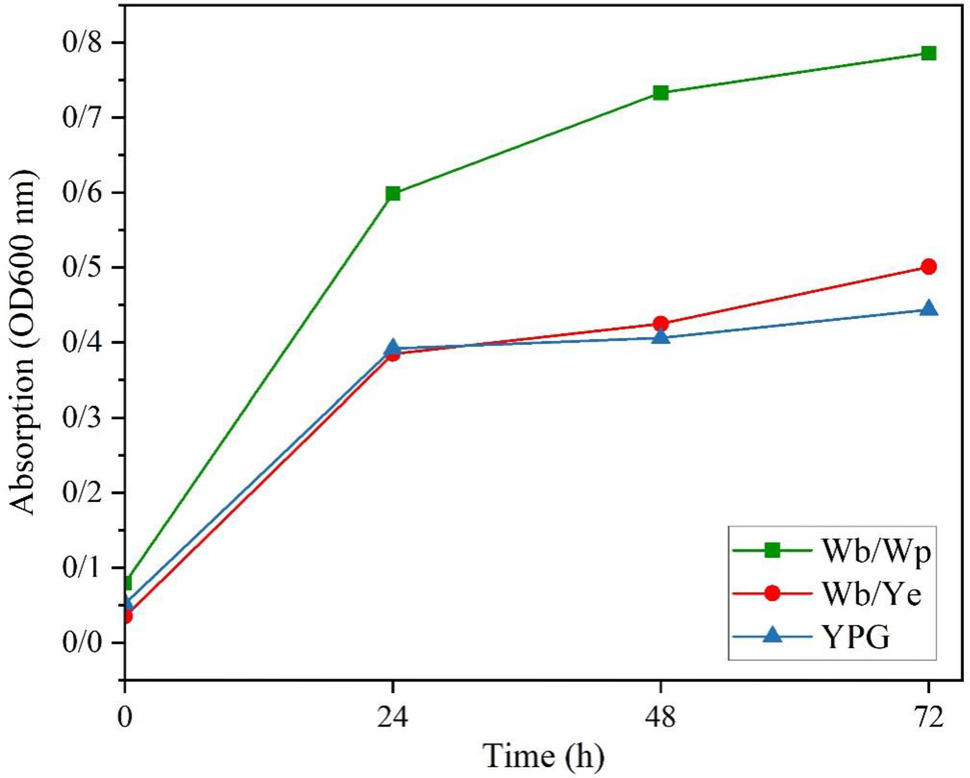
Absorbance analysis of three distinct formulated culture media at 600 nm wavelength post 72-h incubation period.

The culture medium composition plays a pivotal role in yeast growth and expression. Commercial yeast growth mediums typically consist of glucose as a carbon source, peptone, and yeast extract as nitrogen sources. Noteworthy, these cultivation mediums are expensive on the industrial scale^12^. In contrast, lignocellulosic compounds offer an attractive alternative as they are natural, cost-effective, and renewable carbon sources^13^. These compounds have three principal units in their structures: cellulose, hemicellulose, and lignin with cellulose being the primary component interconnected to hemicellulose units by lignin, these compounds are not readily accessible to microorganisms. Consequently, pre-purification stages are required to make monosaccharide of the cellulose and hemicellulose accessible to the microorganisms in the medium^14,16^. This approach can be Facilitate their bioavailability to the microorganisms and making it a cost-effective approach. The initial investment in the pre-treatment processes is mitigated by the low cost of the raw materials and the high nutrient yield, paving the way for widespread application on a large scale. In this study, wheat bran as a lignocellulosic source was purified twice through acid hydrolysis and after filtration that supernatant utilized as the carbon source in two culture mediums, Wb/Ye and Wb/Wp. Remarkably, all three formulated mediums, YPG, Wb/Ye, and Wb/Wp, demonstrated an increase in biomass. The comparison of growth process in three culture medias over 72 h of incubation was monitored by measuring absorbance at OD 600 nm at intervals (0, 24, 48, and 72 h), as depicted in Fig. 1. The yeast biomass growth in all three mediums exhibited an exponential pattern. Interestingly, the Wb/Wp culture medium, with whey protein as the nitrogen source, showed the highest absorbance after 72 h of incubation, indicating a higher yeast cell growth rate in this medium. Overall, the results suggest that the wheat bran pretreatment process is highly effective and makes the glucose in the cellulose and hemicellulose structures available to the growing cells.

### Characterization of disrupted yeast cells

#### Microscope photos

**Figure 2:**
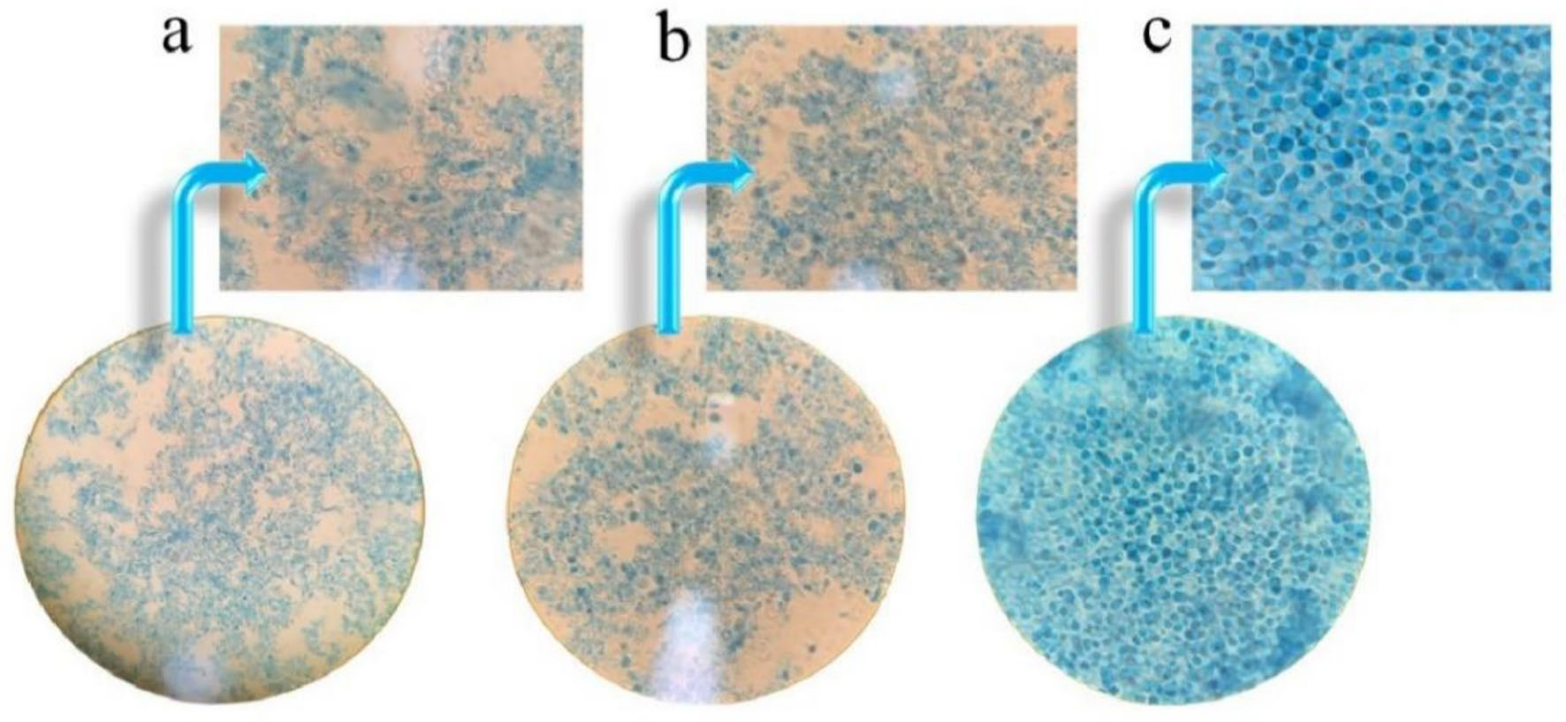
Comparative analysis of yeast cell disruption through various approaches: (**a**) Physical lysis (Ph); (**b**) combined hydrolysis-physical (H-Ph); (**c**) hydrolysis (H).

Figure 2 shows microscopic visualizations that confirm the efficiency of the physical method (Ph) in homogenizing yeast cells, which results in thorough disintegration of the cell wall. This ensures that the internal cell components and organelles are effectively released. Figure 2a,b show clusters of YCW polymers. These clusters highlight the dominant presence of hydrophobic β-glucans, as shown by the studies of Bertsch et al.^17^ and further confirmed by Bzducha-Wróbel et al.^18^. It is therefore obvious that these methods are very suitable for producing fragmented yeast cells and at the same time facilitating the purification of cytosolic elements. In contrast, Fig. 2c shows cells that remain largely intact. This indicates that only a portion of the cells were digested during the hydrolysis process. The lower efficiency of this method and its implications are discussed in more detail in the following sections.

#### Yeast cell disruption analysis

**Figure 3:**
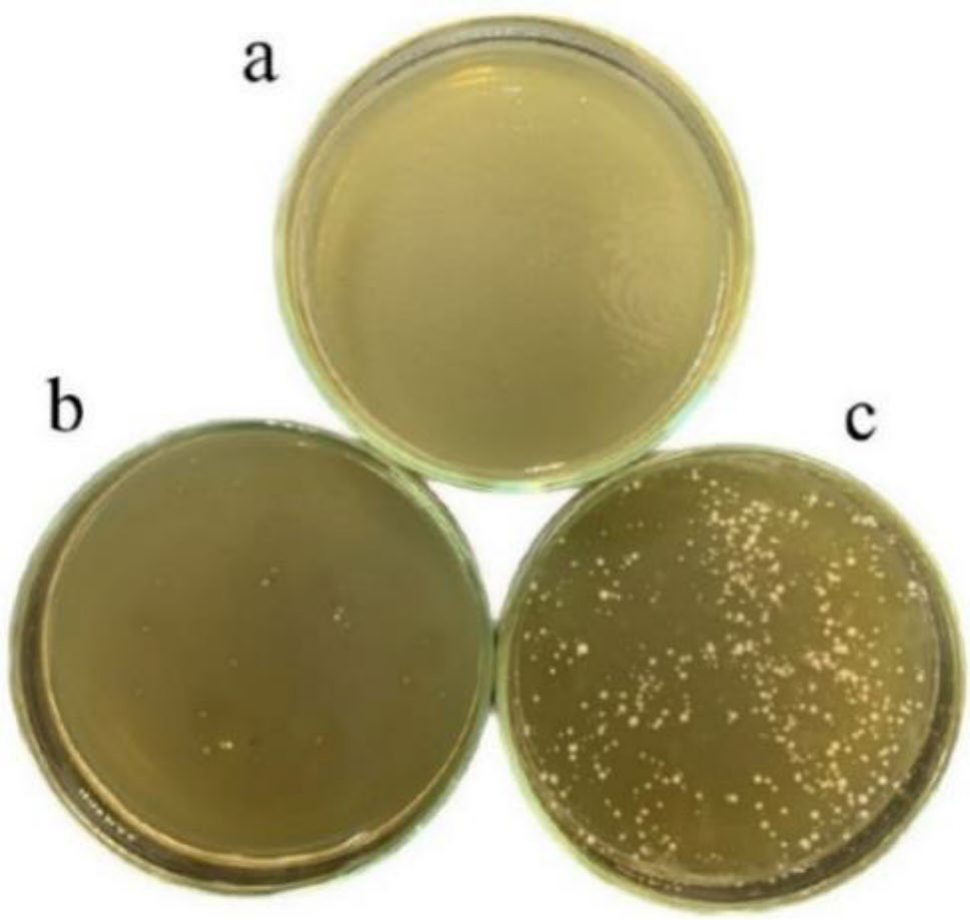
Comparative growth of yeast cells on YPG media after 24-h incubation. Plate (**a**) shows growth after physical lysis (Ph), plate (**b**) after combined hydrolysis-physical lysis (H-Ph) and plate (**c**) after hydrolysis (H).

**Figure 4:**
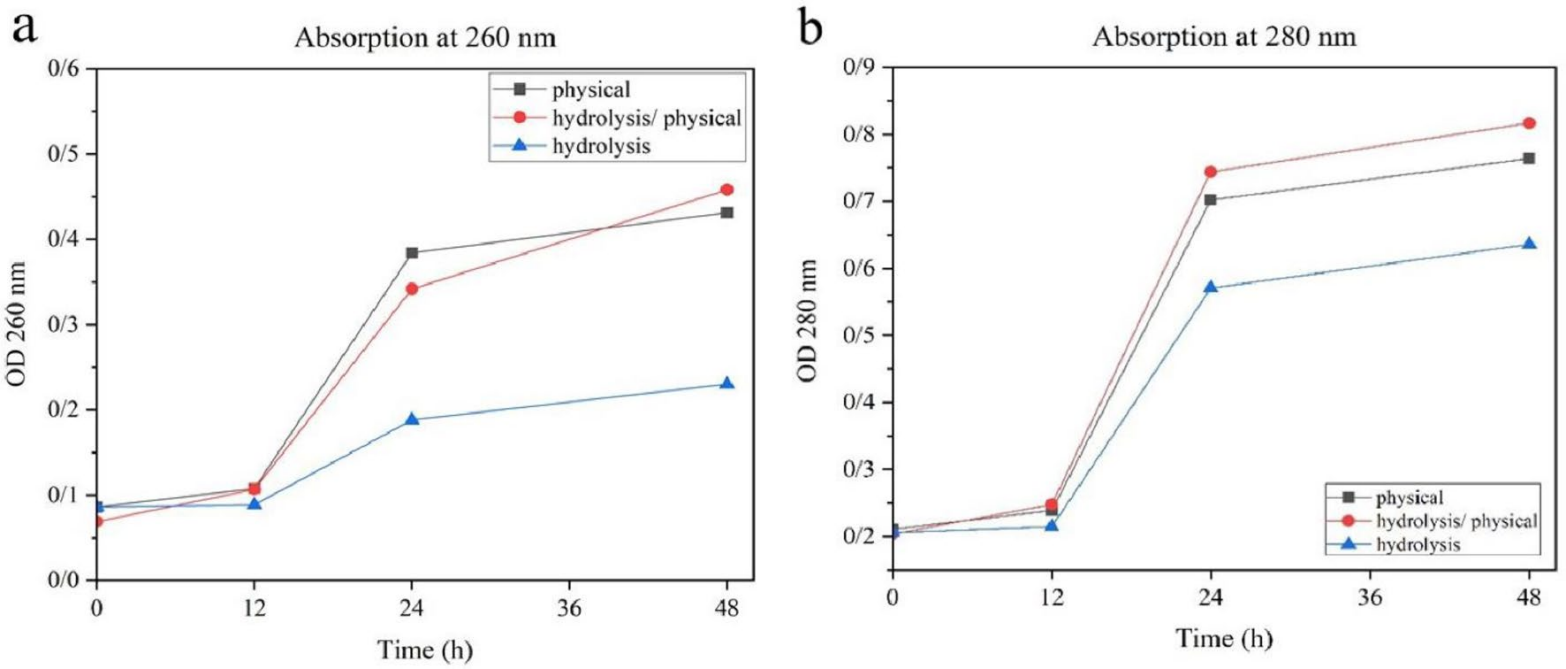
Monitoring cell damage and cytoplasmic component leakage in the supernatant: (**a**) absorbance measured at 260 nm; (**b**) absorbance measured at 280 nm.

The effectiveness of the different disruption methods in releasing intracellular components of yeast cells was evaluated by two different analyzes. In the first analysis, culturing yeast suspensions on agar plates showed significant differences in cell viability after disruption. In the samples where physical lysis was performed, there were almost no surviving cells 48 h after incubation (Fig. 3a,b). In contrast, suspensions obtained by hydrolytic disruption still had living cells after 48 h incubation (Fig. 3c). During analysis, absorbance was measured at OD 260 and 280 nm to monitor the extent of cell disruption and the resulting release of cell contents such as nucleic acids, proteins, and polysaccharides (Fig. 4). The data showed that after 48 h lysis period, samples processed by the Ph method using glass beads had higher absorbance than samples processed just by the H method. At this stage of analysis, the yield of solid residues excreted after disruption was considered an important marker for the release of intracellular space. Synthesis of the observations revealed that the hybrid hydrolytic-physical method is a superior strategy for cell disruption because it allows optimal recovery of intracellular contents compared to the other individual methods. This seems to agree with the findings of Běehalová and Beran^19^ and Takalloo et al.^20^, who indicated that enzymatic hydrolysis mainly attacks the cell wall and facilitates the conversion of proteins into smaller peptides that can leave the cell. Therefore, the combination of pH lysis method and H lysis method may increase the efficiency of the process and allow a higher yield in the extraction of intracellular constituents.

### Effect of analyzed disruption methods on the content of total nitrogen and saccharides in the cell wall preparation

#### Protein release

The release of proteins is a critical aspect of yeast cell disruption procedures^21^. From the Kjeldahl analysis data (see Table 3); the protein content of the cell wall sample obtained by Ph disruption was about 37.49%. In contrast, the H disruption yielded a significantly higher protein content of about 41.65% in the cell wall. Interestingly, both values are higher than the protein content of the yeast cell wall samples obtained by H-Ph disruption, which was about 28.54%. In Ph disruption methods, cell fission leads to the best fragmentation of cells, which helps to effectively release proteins and other intracellular compounds. However, there is a possibility of significant contamination by proteins derived from other cytoplasmic components such as mitochondria and ribosomes^22^.

**Table 3:** Results of the Kjeldahl analysis of cell walls obtained using different disruption methods.

Item	Ph	H/ Ph	H	SEM	P-value
CP (% of DM)	37.49^b^	28.54^a^	41.65^b^	2.571	0.02

**Values with differing letters (a, b) are significantly different.

*Ph* physical lysis, *H-Ph* combined hydrolysis and physical lysis, *H* hydrolysis.

However, in the duration of the disruption method using enzyme (H), the cell wall is only porous, and the hydrolytic activity of the enzyme is gradually steered toward the proteins on the cell surface^20^. Also, this method of disruption is a time-consumer and costly process.

The lowest protein content observed in the cell wall with the H-Ph method compared to the Ph and H methods alone could be due to the initial enzymatic pretreatment phase. This phase could mitigate the introduction of unwanted protein impurities. As noted by Iten and Matile^23^, this enzymatic phase facilitates the partial hydrolysis of extracellular proteins on the cell surface, thereby possibly protein removing gradually occurs from cytoplasm under the influence of the hydrolytic activity of the enzyme^24^. Hence, it can be claimed that partial physical lysis after hydrolytic pretreatment can lead to the completion of cell disruption process, and fragmentation of the cell, as well as the exit of the cell contents and their entry into the extract with the lowest protein contamination of various other intracellular organelles.

#### Saccharide content

**Figure 5:**
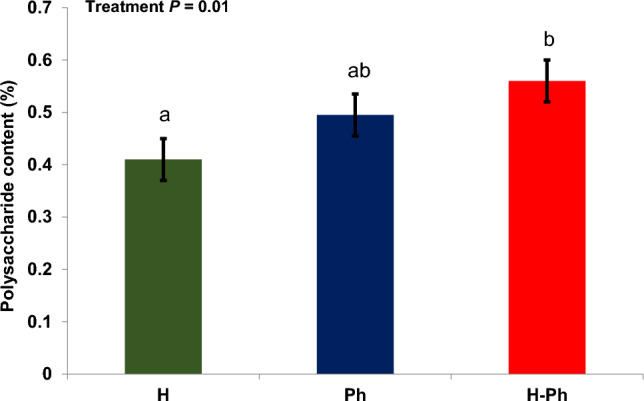
Analysis of sugar content in yeast cell walls derived from various disruption techniques. The lowercase letters (**a**–**c**) indicate significant differences between the treatments. Values are means ± standard error.

Figure 5 shows that the cell wall preparations obtained by the H-Ph disruption method contain an average 56% total saccharides. This figure is consistent with the observed decrease in protein content in the YCW compared to the whole cell. In contrast, the Ph disruption method yielded an average total saccharide content of about 49%. It is worth noting that the use of glass beads with a size between 0.5 and 1 mm in the physical disruption method could lead to similar results in terms of protein and total saccharide content^18^. The H disruption method gave an average total saccharide content of about 41%. During the autolytic or hydrolytic process, the polysaccharides of YCW are gradually degraded. This process alleviates the osmotic pressure in the cell cytoplasm and keeps the cellular polysaccharides largely intact, albeit at the expense of a partial loss of structural polymers in the cell wall. Therefore, the extraction of β-glucans from these cell wall fractions proves to be suboptimal^25^. Considering the higher total saccharide content and lower protein concentration in the cell wall samples obtained by the H-Ph disruption method (compared to the other methods), this cell wall fraction was preferred for β-glucan extraction. This approach appears to ensure comprehensive YCW fragmentation while minimizing protein contamination by other cell organelles.

#### FTIR spectroscopy of the optimal cell wall and extracted β-glucan

**Figure 6:**
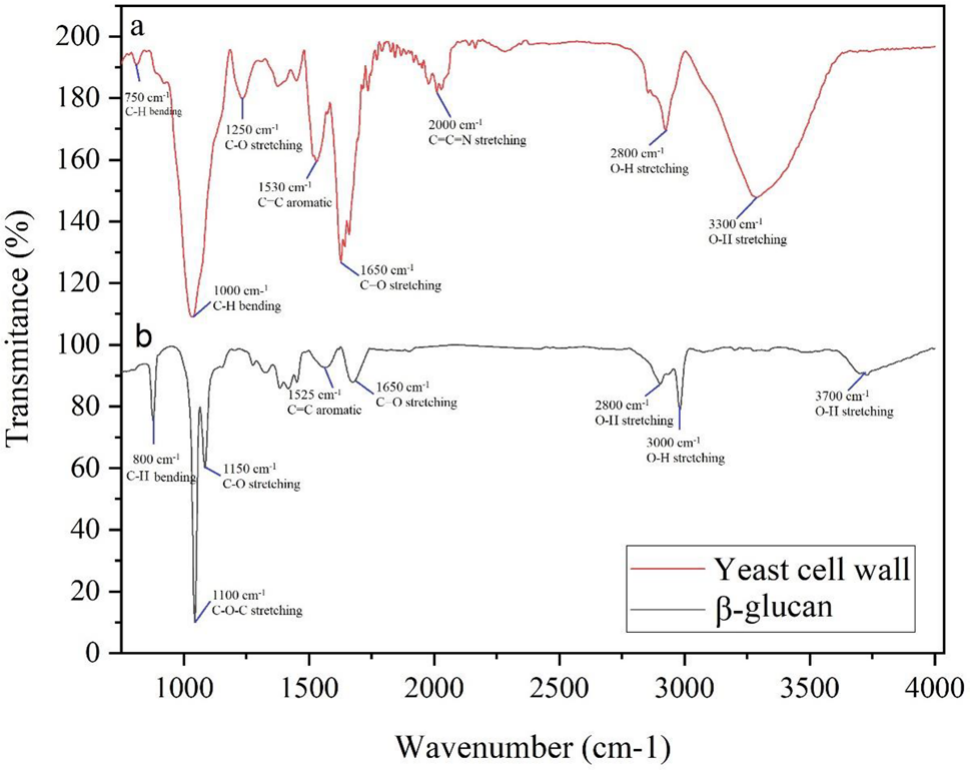
Infrared (IR) spectra of two analytical samples: (**a**) post cell disruption yeast cell wall preparation; (**b**) β-glucan extracted from yeast cell wall.

FTIR analysis was carried out to explore the architecture of the optimal YCW after cell lysis (H-Ph disruption method), and the β-glucan derived from it. Additionally, this analysis allowed for the comparison of structural alterations to the β-glucan post-extraction from the cell wall. The recorded FTIR spectra of two samples are shown in Fig. 6, where “a” represents YCW from the optimal sample after cell lysis and “b” represents extracted β-glucan from the optimal YCW sample. The structural changes due to the conversion of the cell wall to β-glucan can be clearly seen in the graph of “b”. The bands in the 750–950 cm^−1^ and 950–1200 cm^−1^ ranges (C–C, C–O, and C–O–C stretching bands) are characteristic of polysaccharides, which can be seen in the graph of samples “a” and “b”, respectively. These regions represent the anomeric and sugar regions, respectively^20,26^. The bands in the range 1450–1650 cm^−1^ are associated with the stretching vibrations of C=O, C–N and N–H. According to Novák et al.^27^, these bands indicate amide bonds and aromatic rings. The 1650 cm^−1^ band in sample “b” (after extraction of β-glucan from the wall) is smaller than that of sample “a”, which is due to a decrease in protein content and an increase in the purity of saccharides after extraction of β-glucans. In addition, the bands in the 2850–2921 cm^−1^ ranges are indicators of aliphatic C–H groups in pyranoid rings (C–H stretching bands) seen individually in both samples. These observations are consistent with those of Hromádková et al.^28^. Also, the visible bands in the region of 3300 cm^−1^ in sample “a” and 3750 cm^−1^ in sample “b” are indicative of OH stretching vibrations^4^.

#### Antibacterial assay

**Figure 7:**
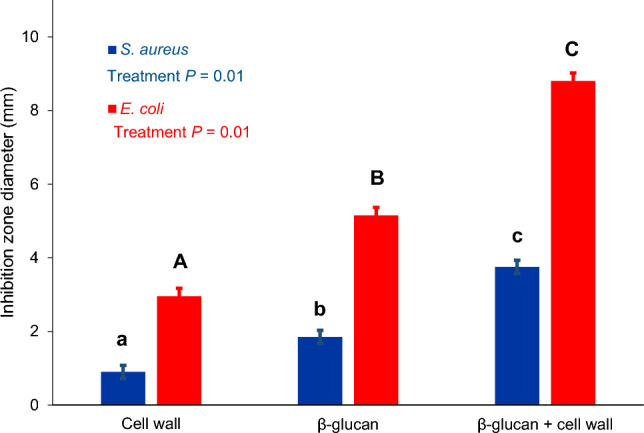
Evaluation of the antibacterial potency of the yeast derivatives studied: remarkable diameter of the inhibition zones formed by the mixture of β-glucan and cell wall. The lowercase letters (a, b, c) indicate significant differences between the treatments for *S. aureus*, while the uppercase letters (A, B, C) indicate significant differences between the treatments for *E. coli.* Values are means ± standard error.

The antibacterial activity of the optimized cell wall, β-glucan, and their combination against the growth of *E. coli* and *S. aureus* is shown in Fig. 7. The combination of extracted β-glucan and YCW had the most pronounced effect on the bacteria studied. This can be attributed to the low molecular weight of the extracted β-glucan, which can easily penetrate the bacteria and disrupt their metabolism. The minimum concentration of the combination of β-glucan and YCW that inhibited the growth of *E. coli* and *S. aureus* was measured to be 250 µg/mL and 500 µg/mL, respectively (Table 4). All results showed that the studied yeast derivatives exhibited stronger antibacterial activity against Gram-negative bacteria than against Gram-positive ones. Our results agree with previous studies^29^. The hardness and resistance caused by the thick peptidoglycan of the Gram-positive bacteria may be the reason^26,30^.

**Table 4:** MIC and MBC values of yeast derivatives investigated against *S. aureus* and *E. coli.*

Bacteria	MIC (µg/mL)		MBC (µg/mL)	
Antibacterial agent	*S. aureus*	*E. coli*	*S. aureus*	*E. coli*
β-Glucan + cell wall	500	250	500	250
β-Glucan	>500	500	>500	500
Cell wall	>500	500	>500	>500

### Adsorbing capacity of studied mycotoxin's binders

The binding efficiency of mycotoxins to YCW adsorbents is significantly influenced by their physical attributes. The YCW is an intricate lattice primarily formed by polysaccharides. It is coated on the outside with richly glycosylated mannoproteins that play a key role in cell recognition and enhance the mycotoxin adsorption mechanism. Additionally, the thickness and composition of the YCW are vital determinants, with increased thickness and higher content correlating with enhanced detoxification capabilities against mycotoxins. Utilization of yeasts and their derivatives has been recognized as a preventive strategy against the detrimental effects of toxins on both human and animal health^31,32^. Within the scope of this discourse, the adsorption mechanism of the YCW is underscored by the critical interaction between β-(1,3)-d-glucans and mycotoxins. The principal interactions facilitating mycotoxin adsorption are Van der Waals forces, which manifest between the aromatic cycles of the mycotoxins and the β-d-glucopyranose rings within the YCW. This is supplemented by hydrogen bonds forming between the hydroxyl, ketone, and lactone functional groups of the mycotoxins and the hydroxyl constituents of the glucose molecules found in the β-D-glucans of the YCW^33^. Moreover, the detoxification potential is modulated by the physical parameters of the YCW, where an increment in wall thickness and biochemical constituency is directly proportional to an increase in mycotoxin detoxification efficacy^34^. As Table 5 shows, when AFB1 was absorbed by two developed binders, the mixture of β-glucan and YCW was assigned a higher absorption capacity (P < 0.05). Apparently, the hydroxyl, ketone, phenyl, and lactone groups in β-glucans are involved in the formation of hydrogen and van der Waals bonds between AFB1 and glucan, and the increased groups in a mixture of β-glucan and YCW could explain the observed results^11,35^. The YCW used as a binder for AFB1 showed the lowest binding rate and did not exceed 50%. Despite this observation, a significant difference was observed between the absorption properties of YCW and the combination of β-glucan and YCW in AFB1 absorption (P < 0.05). In agreement with our results on the binding of AFB1 to the YCW and its components, Hojati et al.^35^. reported that YCW products have an average efficiency in AFB1 absorption in vitro. According to the results of OTA absorption by the tested binders, it was also shown that the mixture of β-glucan with the cell wall had a higher absorption capacity for this toxin (P < 0.05). Bornet and Tessedre^36^ showed that chitin and β-glucan mixtures, as well as wall hydrolysis, have a relatively high ability (64–74%) to remove OTA from OTA-contaminated wine. In addition, Piotrowska and Masek^4^ showed that YCW components, especially glucan extracted from them, are responsible for OTA adsorption at nearly neutral pH.

**Table 5:** The mycotoxins adsorption ability of the yeast-derived products.

Adsorbents	*Aflatoxin B1* (%)	*Ochratoxin A* (%)	*Zearalenone* (%)	SEM	P-value
Cell wall	47.16^a^	52.96^ab^	61.10^b^	3.421	0.04
Cell wall + β-glucan	60.70^a^	65.85^a^	80.85^b^	4.813	0.01

**Numbers with same letters have not significant differences.

As the observed results on ZEN adsorption show, the absorption of this mycotoxin by two types of binders was higher than that of other mycotoxins. Among the components of the YCW, β-glucans are the main molecules responsible for the absorption of ZEN, and the absorption capacity of YCW strongly depends on its β-glucan content^5^. Generally, the properties of adsorbed toxins such as polarity, solubility and charge distribution play an important role in the binding process and are manifested in various binding mechanisms such as non-covalent interactions, hydrogen bonding, etc^37,38^. ZEN is a macrocyclic molecule and much more hydrophobic than aflatoxin. The presence of diphenolic groups made it a weak acid. Consequently, the adsorbent must also have polarity in order for the bond between ZEN and the binder to be properly established^2^. A structural change in the composition of the β-glucan when these two molecules come into contact with each other enhances the binding process^5^.

Of the two types of our binders, the highest absorption rate for ZEN is associated with the mixture of β-glucan and YCW, and in general this mixture can be chosen as the optimal binder among the other binders used. The stronger binding of these binders can be justified by the report of Yiannikouris et al.^5^. According to this report, the first bound molecules of ZEN open the helix structure of β-glucans and thus bind more molecules of the toxin. In general, various functional groups in β-glucans, including C=O, O–H, C–O–C, C–C, etc., have high absorption capacity for various mycotoxins. On the other hand, the combination of pure β-glucan with YCW, which also contains a few polysaccharides such as mannan-oligosaccharides, β-glucans, and chitin, increases the absorption of mycotoxins. This is because the combination of these two binders brings a greater diversity of functional groups that cause the formation of different bonds with mycotoxins. Therefore, the combination of the YCW with β-glucan is likely to increase the amount of glucan accessible to the toxins and consequently cause higher binding.

## Conclusion

In this study, we have developed innovative methods for the extraction of yeast derivatives, in particular yeast cell wall and β-glucan. Using a technique that combines physical lysis with enzymatic pretreatment for cell disruption, we achieved a product with a high polysaccharide concentration and minimal protein content. After the successful extraction of β-glucan, we investigated the mycotoxin adsorption capacity of both the cell wall and the β-glucan, separately and in combination. Our results indicate that a composite of cell wall and β-glucan exhibited significantly improved mycotoxin adsorption performance, surpassing the adsorptive properties of each component when used individually. In particular, the enhanced ability to adsorb mycotoxins was most pronounced for *zearalenone* when it was bound to this composite. We hypothesize that this increased affinity may be due to intrinsic factors in the glucans, such as the presence of various chemical groups that may alter the interaction dynamics with mycotoxins. In addition, the inherent antibacterial activity of the composite, especially against Gram-negative bacteria, increases its potential as a nutritious additive for human and animal consumption. Overall, the findings obtained in the study on the robust adsorption properties of the glucan-cell wall composite underscore its suitability as a dietary supplement, which is crucial for strengthening health and protecting against the penetration of mycotoxins.

## Data Availability

The datasets used and/or analysed during the current study are available from the corresponding author on reasonable request.
